# Ocular Complications During Prone Positioning Ventilation in Critically Ill Adults: A Systematic Review

**DOI:** 10.1002/nop2.70770

**Published:** 2026-08-21

**Authors:** Zongheng Jiang, Peipei Gu, Jianming Wang, Peng He, Yue Jiang, Fei Zeng

**Affiliations:** ^1^ Department of Nursing The Second Affiliated Hospital, Zhejiang University School of Medicine Hangzhou Zhejiang Province China

**Keywords:** critical care, eye care, mechanical ventilation, ocular complications, prone position, systematic review

## Abstract

**Aim:**

To synthesise ocular outcomes, assessment methods and associated factors among critically ill adults exposed to prone positioning ventilation.

**Design:**

Systematic review with narrative synthesis.

**Review Methods:**

Two reviewers independently completed study selection, data extraction and methodological appraisal. Findings were synthesised narratively because heterogeneity precluded meta‐analysis.

**Data Sources:**

PubMed, Embase, CINAHL, Web of Science and the Cochrane Library were searched from inception to November 2025, with backward citation searching.

**Results:**

Seven studies involving 722 critically ill adults were included. Reported findings included chemosis, exposure keratopathy, conjunctival abnormalities and increased intraocular pressure. Chemosis and exposure keratopathy were the most frequent ocular surface manifestations. Two studies evaluated physiological increases in intraocular pressure rather than clinical complications, and several mixed intensive care cohorts lacked prone‐position‐specific estimates. Reported associated factors included lagophthalmos, deeper sedation, higher positive end‐expiratory pressure, prolonged or repeated prone positioning and increased ocular secretions, although multivariable evidence was limited.

**Conclusion:**

Critically ill adults exposed to prone positioning may develop ocular abnormalities, but heterogeneous designs, assessment methods, and outcome reporting prevent reliable estimation of incidence.

**Implications for the Profession and/or Patient Care:**

Ocular risk assessment should be integrated before, during, and after prone positioning. Preventive eye care, repeated assessment, documentation, and timely ophthalmological referral may support early identification and reduce avoidable injury.

**Impact:**

This review addresses fragmented evidence on ocular safety during prone positioning, identifies common ocular findings and highlights clinically relevant associated factors. The findings are relevant to critical care nurses and prone‐positioned adults.

**Reporting Method:**

The review followed the Preferred Reporting Items for Systematic Reviews and Meta‐Analyses 2020 statement.

**No Patient or Public Contribution:**

Patients and the public were not involved because this review analysed published studies.

**Trial and Protocol Registration:**

International Prospective Register of Systematic Reviews, CRD420251268507.

## 1 Background

Critically ill adults in intensive care units may require prone positioning ventilation for moderate‐to‐severe acute respiratory distress syndrome (Guérin et al. 2013; Langer et al. 2021). Although this intervention improves ventilation–perfusion matching, promotes dorsal lung recruitment and reduces mortality, it may also expose patients to treatment‐related complications, including ocular injury (Binda et al. 2021; González‐Seguel et al. 2021; Guérin et al. 2013). Ocular complications in this context may include ocular surface and anterior segment abnormalities, such as chemosis, exposure keratopathy, conjunctival haemorrhage and corneal epithelial injury, as well as physiological changes such as increased intraocular pressure. These outcomes should be distinguished from transient facial or periorbital oedema alone, although oedema may contribute to subsequent ocular surface exposure and injury.

Critically ill patients are already vulnerable to ocular surface damage because sedation, neuromuscular blockade, reduced blinking and incomplete eyelid closure impair normal tear‐film distribution and corneal protection (Grixti et al. 2012; Hearne et al. 2018; Rosenberg and Eisen 2008). Prone positioning may further increase this vulnerability through dependent facial oedema, impaired venous return, prolonged ocular exposure and direct pressure on the orbit (Kwee et al. 2015; Sanghi et al. 2021). Previous studies have reported chemosis, exposure keratopathy and increased intraocular pressure among patients receiving prone positioning (Bajwa et al. 2010; Ceylan et al. 2022; Deol et al. 2020; Han et al. 2025; Saran et al. 2019). However, the available evidence is difficult to interpret because studies have used different populations, ocular assessment methods, outcome definitions and observation time points. Some studies have reported clinical ocular complications, whereas others have focused on physiological changes in intraocular pressure. In addition, several studies included mixed intensive care populations without reporting separate outcomes for patients who underwent prone positioning.

A previous systematic review compared ocular injury between prone and supine positioning, but limited information was available regarding the spectrum of ocular findings, bedside assessment methods and potentially modifiable clinical factors (Patterson et al. 2021). Evidence relating to prone‐position duration, eyelid closure, sedation and ventilation parameters also remains fragmented, and these factors have not been evaluated consistently across studies (Han et al. 2025; Kousha et al. 2018). Therefore, this systematic review aimed to synthesise the reported ocular outcomes, assessment methods and factors associated with ocular abnormalities among critically ill adults exposed to prone positioning ventilation.

## 2 Methods

This systematic review was conducted with reference to the Cochrane Handbook for Systematic Reviews of Interventions and reported in accordance with the Preferred Reporting Items for Systematic Reviews and Meta‐Analyses (PRISMA) 2020 statement (Higgins et al. 2019; Page et al. 2021).

### 2.1 Eligibility Criteria

The eligibility criteria were developed using the PICOS framework. The population of interest comprised critically ill adults aged 18 years or older who were treated in intensive care units. Studies were eligible if all participants received prone positioning ventilation or if prone positioning was explicitly reported or examined as a clinical exposure within a mixed intensive care cohort.

The exposure of interest was prone positioning ventilation, including single or repeated prone sessions, without restriction on session duration. A comparator group was not mandatory because several eligible studies used single‐group observational or before‐and‐after designs.

Eligible study designs included randomised controlled trials, quasi‐experimental studies, prospective or retrospective cohort studies, cross‐sectional studies, case–control studies, and case series that provided extractable ocular outcome data.

Studies were excluded if they:

1. involved paediatric patients or patients outside intensive care settings;
2. did not report ocular findings or intraocular pressure outcomes relevant to prone positioning;
3. were single case reports, reviews, expert opinions, editorials, protocols or conference abstracts; or
4. were not published in English.

For mixed populations, only data relating to critically ill adults were extracted. When a study included both prone and non‐prone patients but did not report a prone‐position‐specific denominator, the overall intensive care cohort findings were retained only when prone positioning was explicitly reported or analysed as a relevant clinical exposure. Such findings were clearly identified as non‐prone‐specific in the synthesis.

### 2.2 Outcomes of Interest

The primary outcomes were:

1. the reported frequency or proportion of ocular findings;
2. the types of ocular complications or abnormalities identified during intensive care or prone positioning; and
3. changes in intraocular pressure before, during, or after prone positioning.

Ocular outcomes included chemosis, exposure keratopathy, conjunctival hyperaemia, corneal epithelial injury, subconjunctival haemorrhage, periorbital oedema, ocular discharge and other clinically reported ocular events.

The secondary outcome was any factor statistically examined in relation to ocular findings or intraocular pressure changes, including prone positioning duration or frequency, eyelid closure status, sedation, mechanical ventilation parameters, and other clinical exposures.

### 2.3 Search Strategy

PubMed, Embase, CINAHL, Web of Science and the Cochrane Library were systematically searched from inception to November 2025. The search strategy was developed with the support of medical librarians using controlled vocabulary and free‐text terms related to prone positioning, ocular complications and intensive care.

The complete search strategy for each database is presented in Table 1. Backward citation searching of the reference lists of included studies and relevant reviews was also undertaken to identify additional records.

**TABLE 1:** Search strategy.

Database	Index words	Results
PubMed	(((Eye Injuries[MeSH Terms]) OR (Vision Disorders[MeSH Terms]) OR (Ocular Hypertension[MeSH Terms]) OR (Corneal Injuries[MeSH Terms]) OR (Optic Neuropathies[MeSH Terms]) OR (Periorbital Dermatitis[MeSH Terms]) OR (“eye complication”[Title/Abstract]) OR (“ocular complication”[Title/Abstract]) OR (“corneal injury”[Title/Abstract]) OR (“increased intraocular pressure”[Title/Abstract]) OR (“central retinal artery occlusion”[Title/Abstract]) OR (“vision loss”[Title/Abstract])) AND ((prone position[MeSH Terms]) OR (“prone position”[Title/Abstract]) OR (“prone ventilat*”[Title/Abstract]) OR (“prone posture”[Title/Abstract])) AND ((Intensive Care Units[MeSH Terms]) OR (“ICU”[Title/Abstract]) OR (“intensive care”[Title/Abstract]) OR (“critical care”[Title/Abstract]) OR ((Respiration[MeSH Terms]) AND (Mechanical Ventilation[MeSH Terms])) OR (“mechanical ventilation”[Title/Abstract])))	2
CINAHL	#1 ( MH “Prone Position” ) OR TI ( “prone position*” OR “prone ventilat*” OR proning ) OR AB ( “prone position*” OR “prone ventilat*” OR proning ) #2 ( MH “Eye Injuries+” ) OR ( MH “Vision Disorders+” ) OR ( MH “Corneal Injuries” ) OR ( MH “Ocular Hypertension” ) OR ( MH “Optic Neuropathy” ) OR TI ( “eye complication*” OR “ocular complication*” OR “corneal injur*” OR “corneal abrasion*” OR “exposure kerat*” OR “ischemic optic neuropath*” OR “increased intraocular pressure” OR “vision loss” OR “periorbital edema” OR chemosis ) OR AB ( “eye complication*” OR “ocular complication*” OR “corneal injur*” OR “corneal abrasion*” OR “exposure kerat*” OR “ischemic optic neuropath*” OR “increased intraocular pressure” OR “vision loss” OR “periorbital edema” OR chemosis ) #3 #1 AND #2 #4 ( MH “Intensive Care Units+” ) OR ( MH “Critical Care+” ) OR TI ( ICU OR “intensive care” OR “critical care” ) OR AB ( ICU OR “intensive care” OR “critical care” ) #5 ( MH “Mechanical Ventilation+” ) OR TI ( “mechanical ventilat*” OR ventilat* ) OR AB ( “mechanical ventilat*” OR ventilat* ) #6 #4 OR #5 #7 #3 AND #6	9
Web of Science	(“prone position*” OR “prone ventilat*” OR proning)AND(“eye complication*” OR “ocular complication*” OR “corneal abrasion*” OR “exposure kerat*” OR “ischemic optic neuropath*” OR “increased intraocular pressure” OR “vision loss” OR “periorbital edema”)AND(“intensive care unit*” OR ICU OR “critical care” OR “mechanical ventilat*”)	118
Embase	#1 'prone position'/exp OR 'prone position*':ti,ab,kw OR 'prone ventilat*':ti,ab,kw OR proning:ti,ab,kw #2 'eye injury'/exp OR 'visual disorder'/exp OR 'exp cornea injury' OR 'ischemic optic neuropathy'/exp OR 'ocular hypertension'/exp OR 'retina artery occlusion'/exp #3 #1 AND #2 #4 'intensive care'/exp OR 'critical care'/exp OR icu:ti,ab,kw OR 'intensive care':ti,ab,kw OR 'critical care':ti,ab,kw #5 'artificial ventilation'/exp OR 'mechanical ventilat*':ti,ab,kw OR ventilat*:ti,ab,kw #6 #4 OR #5 #7 #3 AND #6 #8 #7 AND 'human'/de	87
Cochrane Library	#1 (prone next position*) or “prone ventilat*” or proning(ti,ab,kw) #2 (eye complication*) or (ocular complication*) or (corneal abrasion*) or (exposure kerat*) or (ischemic optic neuropath*)(ti,ab,kw) #3 #1 and #2	5

### 2.4 Study Selection

All retrieved records were imported into reference‐management software, and duplicate records were removed. Study selection was undertaken in three stages: title screening, abstract screening, and full‐text eligibility assessment.

**FIGURE 1:**
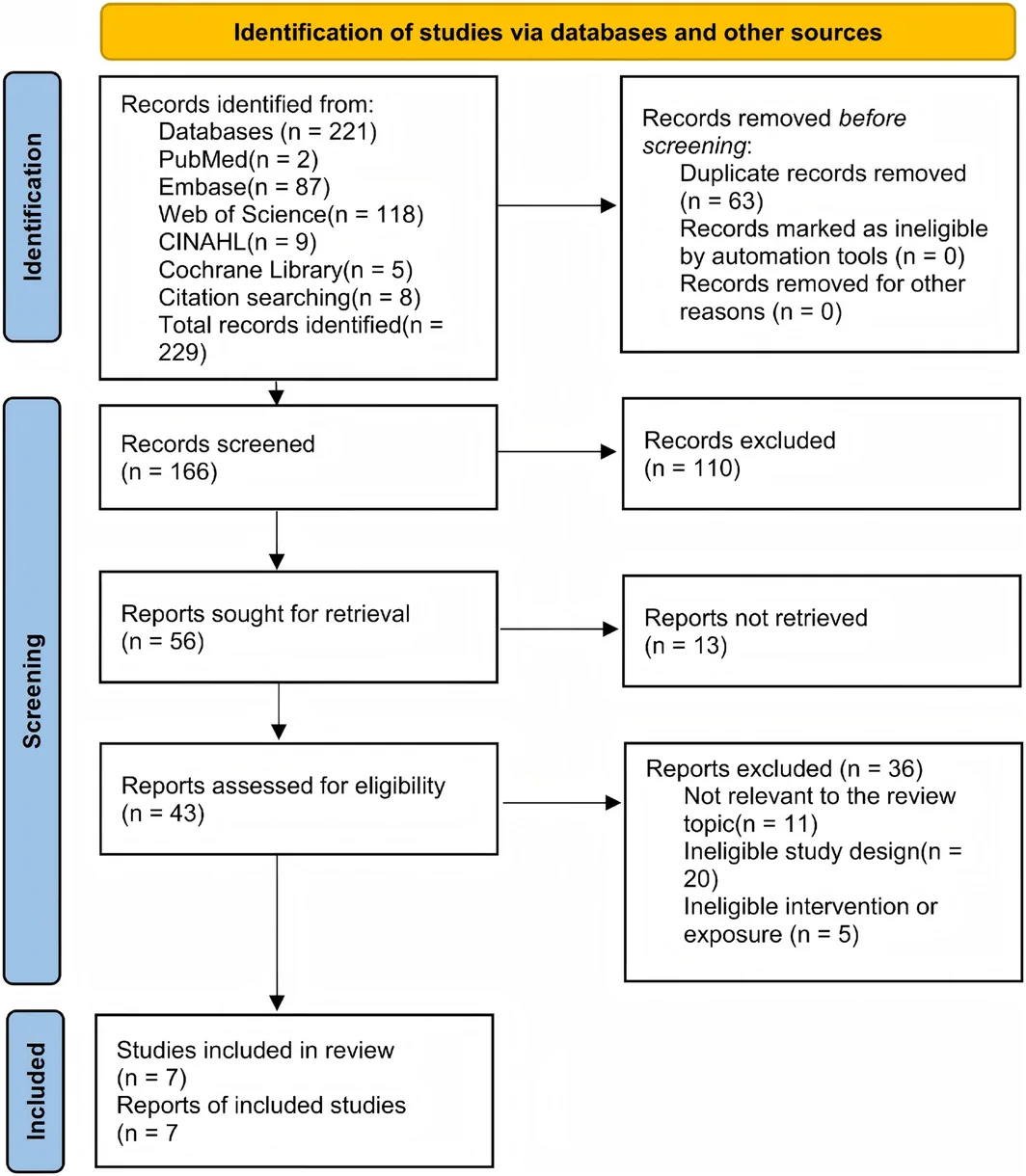
PRISMA 2020 flow diagram of the study selection process.

Two reviewers independently screened the records against the eligibility criteria. Disagreements were resolved through discussion, with consultation from a third reviewer when consensus could not be reached. The study‐selection process is presented in the PRISMA 2020 flow diagram in Figure 1.

### 2.5 Data Extraction

Data were extracted using a predefined data‐extraction form. Extracted information included:

- author and publication year;
- country;
- study design and sample size;
- patient characteristics;
- exposure to prone positioning;
- ocular assessment methods;
- reported ocular findings;
- frequencies, proportions or intraocular pressure changes; and
- factors statistically examined in relation to ocular outcomes

One reviewer performed the initial extraction, and a second reviewer cross‐checked all extracted data against the original publications. Discrepancies were resolved through discussion.

### 2.6 Quality Appraisal

Methodological quality was assessed using design‐specific appraisal tools. Cross‐sectional studies were evaluated using the Joanna Briggs Institute critical appraisal checklist for studies reporting prevalence data (Moola et al. 2020). Cohort and retrospective record‐review studies were assessed using the Newcastle–Ottawa Scale (Wells et al., n.d.). The non‐comparative study was evaluated using the Methodological Index for Non‐Randomised Studies instrument (Slim et al. 2003).

Two reviewers independently completed the quality appraisal. Any disagreements were resolved through discussion or consultation with a third reviewer. Quality appraisal results were used to inform interpretation of the evidence and were not used as exclusion criteria.

### 2.7 Data Synthesis

The findings were synthesised narratively and organised according to study characteristics, prone‐position exposure, ocular assessment methods, reported ocular outcomes and associated factors.

A meta‐analysis was not undertaken because of substantial clinical and methodological heterogeneity across the included studies. The studies differed in population characteristics, study design, prone‐position exposure, ocular outcome definitions, assessment methods, measurement time points and forms of outcome reporting. Some studies reported proportions of clinical ocular findings, whereas others evaluated continuous changes in intraocular pressure. Statistical pooling of these heterogeneous outcomes was therefore considered inappropriate.

Before synthesis, the baseline ocular status and outcome definitions of each included study were re‐examined. The term incidence was used only when the original study confirmed that the outcome was absent at baseline and subsequently recorded newly occurring events during follow‐up. When baseline ocular status was unclear, findings were reported as observed frequencies or proportions rather than incidence estimates. Changes in intraocular pressure were synthesised separately from clinical ocular complications.

Findings from studies exclusively involving patients receiving prone positioning were distinguished from those derived from mixed intensive care cohorts without a prone‐position‐specific denominator. Factors were described as statistically associated with ocular outcomes only when supported by an analysis reported in the original study.

## 3 Results

### 3.1 Studies Included in the Review

A total of 229 records were identified through database and other sources. After duplicate removal, title and abstract screening, and full‐text assessment, seven studies met the eligibility criteria and were included in the review. The study selection process is shown in the PRISMA 2020 flow diagram (Figure 1).

### 3.2 Study Characteristics

The seven included studies involved a total of 722 critically ill adults and were published between 2010 and 2025. The studies were conducted in Türkiye (*n* = 2), the United Kingdom (*n* = 1), China (*n* = 1), the United States (*n* = 1), Portugal (*n* = 1) and India (*n* = 1).

The included studies comprised three prospective observational studies, two cross‐sectional studies, one retrospective record‐review study and one retrospective case series. Four studies directly evaluated ocular outcomes in patients receiving prone positioning ventilation (Bajwa et al. 2010; Deol et al. 2020; Han et al. 2025; Saran et al. 2019). The remaining three studies included mixed intensive care populations in which prone positioning was used or recorded, but prone‐position‐specific denominators or outcome estimates were not reported (Carvalho et al. 2023; Ceylan et al. 2022; Ekici Gok et al. 2021).

Ocular assessment methods differed considerably across studies. These included routine bedside observation, medical‐record review, portable slit‐lamp or biomicroscopic examination, corneal fluorescein staining and tonometry. The reported outcomes included chemosis, exposure keratopathy, conjunctival hyperaemia, punctate keratopathy, subconjunctival haemorrhage, periorbital oedema, ocular discharge and changes in intraocular pressure. Detailed study characteristics and ocular outcomes are presented in Table 2.

**TABLE 2:** Characteristics, ocular assessment methods and reported outcomes of the included studies.

Study, country	Design and sample	Prone‐position exposure	Ocular assessment	Main ocular outcomes	Reported frequency or change	Associated factors
Bajwa et al. (2010), USA	Retrospective case series; *n* = 17, ARDS	Direct; automated prone positioning with axial rotation	Bedside observation	Chemosis	Chemosis observed in 17/17 patients	Not analysed
Saran et al. (2019), India	Prospective observational study; *n* = 25, ARDS	Direct; measurements before, during and after prone positioning	Hand‐held applanation tonometry	Increased IOP	Median intraocular pressure in the non‐dependent eye increased from 14 mmHg before prone positioning to 32 mmHg at the end of the prone session	IOP increased with prone‐position duration; no adjusted analysis
Deol et al. (2020), UK	Prospective observational study; *n* = 12, COVID‐19	Direct; approximately 16 h of prone positioning	Tonometry before and after prone positioning	Increased IOP	Dependent‐eye IOP increased from 11.08 ± 2.57 to 15.58 ± 3.65 mmHg	Not analysed
Ekici Gok et al. (2021), Türkiye	Cross‐sectional study; *n* = 93, COVID‐19 ICU patients	Mixed cohort; prone subgroup denominator unavailable	Portable biomicroscopy and ophthalmoscopy	Chemosis, corneal abrasion, conjunctivitis, retinal haemorrhage and exposure keratitis	Descriptive findings only; no prone‐specific estimate	No adjusted analysis
Ceylan et al. (2022), Türkiye	Prospective observational study; *n* = 70, COVID‐19 ICU patients	Mixed cohort; prone exposure recorded	Daily bedside assessment; ophthalmological assessment when indicated	Chemosis, conjunctival hyperaemia, punctate keratopathy, periorbital oedema and ocular discharge	≥ 1 ocular finding in 59/70 patients (84.3%); no prone‐specific estimate	Invasive mechanical ventilation was associated with chemosis (*p* < 0.001)
Carvalho et al. (2023), Portugal	Retrospective record‐review study; *n* = 253, COVID‐19 ICU patients	Mixed cohort; prone denominator unavailable	Medical‐record review and ophthalmology consultations	Conjunctival haemorrhage, exposure keratitis, conjunctivitis and rare severe events	Ocular complications in 24/253 patients (9.5%); no prone‐specific estimate	Not analysed
Han et al. (2025), China	Cross‐sectional study; *n* = 252, prone‐ventilated patients	Direct; all patients received prone positioning	Corneal fluorescein staining and cobalt‐blue slit‐lamp examination	Exposure keratopathy	Exposure keratopathy in 129/252 patients (51.2%)	Deeper sedation, higher PEEP, longer and more frequent prone positioning, lagophthalmos and increased ocular secretions

*Note:* Reported proportions and IOP changes represent different outcome measures and should not be interpreted as directly comparable incidence estimates. Associated factors are presented only when statistical analysis was reported.

Abbreviations: ARDS, acute respiratory distress syndrome; IOP, intraocular pressure; PEEP, positive end‐expiratory pressure.

Methodological quality ranged from low–moderate to high. Common limitations included small sample sizes, non‐comparative study designs, inconsistent ocular assessment methods, absence of standardised baseline eye examinations and incomplete reporting of potentially relevant clinical variables. The results of the methodological quality appraisal are presented in Tables S1–S3.

### 3.3 Reported Ocular Outcomes

Because the absence of ocular abnormalities before prone positioning or at study enrolment was not consistently documented, the study‐specific findings are presented as observed proportions, frequencies or physiological changes rather than as directly comparable incidence estimates.

Among studies directly examining patients receiving prone positioning ventilation, Han et al. (2025) identified exposure keratopathy in 129 of 252 patients (51.2%) using corneal fluorescein staining and cobalt‐blue slit‐lamp examination. Bajwa et al. (2010) observed chemosis in all 17 patients receiving automated prone positioning and axial rotation; however, the absence of chemosis before the intervention was not systematically documented.

Two studies assessed changes in intraocular pressure rather than the frequency of clinical ocular complications. Saran et al. (2019) reported a progressive increase in intraocular pressure during prone positioning in patients with acute respiratory distress syndrome. Deol et al. (2020) also observed an increase in intraocular pressure following a prolonged prone session, with a more pronounced change in the dependent eye. These findings represent physiological changes during prone positioning and should not be interpreted as complication incidence estimates.

In the mixed intensive care cohorts, Ceylan et al. (2022) reported at least one pathological ocular finding in 59 of 70 patients (84.3%), including conjunctival hyperaemia, chemosis, punctate keratopathy, periorbital oedema and ocular discharge. However, the study did not provide a separate estimate for patients who underwent prone positioning. Carvalho et al. (2023) documented ocular complications in 24 of 253 patients (9.5%) during intensive care admission, but likewise did not report a prone‐position‐specific denominator. Ekici Gok et al. (2021) described chemosis, corneal abrasion, conjunctivitis, retinal haemorrhage and exposure‐related keratitis in critically ill patients with COVID‐19, although a prone‐position‐specific frequency was not available.

Most reported ocular findings involved the ocular surface or anterior segment. Chemosis and exposure keratopathy were the most frequently reported manifestations (Bajwa et al. 2010; Carvalho et al. 2023; Ceylan et al. 2022; Ekici Gok et al. 2021; Han et al. 2025). Less common but clinically severe events, including eyelid ulceration, rhino‐orbital mucormycosis, necrotising scleritis and ocular rupture, were reported in one retrospective ICU study (Carvalho et al. 2023). These rare events were reported for the overall cohort and were not demonstrated to be directly attributable to prone positioning.

### 3.4 Reported Associated Factors

Formal statistical analysis of factors associated with ocular outcomes was limited across the included studies.

In the multivariable analysis conducted by Han et al. (2025), deeper sedation, higher positive end‐expiratory pressure, longer prone positioning duration, more frequent prone positioning sessions, lagophthalmos, and increased ocular secretions were independently associated with exposure keratopathy. Among these factors, lagophthalmos represented a direct indicator of ocular surface exposure, whereas sedation and ventilation‐related variables should be interpreted as clinical conditions that may contribute to impaired eyelid closure or ocular‐surface vulnerability rather than as direct causes of injury.

Ceylan et al. (2022) reported a statistically significant association between invasive mechanical ventilation and chemosis. However, prone positioning was not reported as an independently associated factor, and the study did not provide a prone‐position specific subgroup analysis.

Saran et al. (2019) demonstrated that intraocular pressure increased over the duration of prone positioning, while Deol et al. (2020) reported a greater post‐prone increase in the dependent eye. These findings describe temporal and positional changes in intraocular pressure rather than independently adjusted risk factors.

The remaining studies did not conduct formal statistical analyses of associated factors (Bajwa et al. 2010; Carvalho et al. 2023; Ekici Gok et al. 2021). Although sedation, neuromuscular blockade, mechanical ventilation, and systemic illness were described as relevant clinical exposures in some reports, the available data did not establish these variables as independent causes of ocular complications.

## 4 Discussion

This systematic review identified a range of ocular findings among critically ill adults exposed to prone positioning, including chemosis, exposure keratopathy, conjunctival abnormalities and increased intraocular pressure. Chemosis and exposure keratopathy were the most frequently reported ocular surface manifestations, whereas two studies primarily evaluated physiological changes in intraocular pressure rather than clinical ocular complications (Bajwa et al. 2010; Carvalho et al. 2023; Ceylan et al. 2022; Deol et al. 2020; Ekici Gok et al. 2021; Han et al. 2025; Saran et al. 2019). However, the available evidence was limited and heterogeneous. Differences in study populations, ocular assessment methods, measurement time points and outcome definitions meant that the reported proportions and physiological changes were not directly comparable. In addition, several studies involved mixed intensive care populations and did not provide prone‐position‐specific outcome estimates. The findings should therefore be interpreted as a descriptive synthesis of the available evidence rather than as a pooled estimate of the incidence of prone‐position‐associated ocular complications.

Ocular complications observed during prone positioning should not be considered in isolation from the underlying risks associated with critical illness and mechanical ventilation. Sedated and mechanically ventilated patients are already vulnerable to ocular surface injury because of reduced blink frequency, impaired tear‐film distribution, incomplete eyelid closure, and limited ability to report ocular discomfort (Grixti et al. 2012; Hearne et al. 2018; Jammal et al. 2012; Kousha et al. 2018; Rosenberg and Eisen 2008). Neuromuscular blocking agents may further reduce orbicularis oculi tone and impair eyelid closure. Mechanical ventilation, fluid imbalance, vasopressor use, and systemic inflammation may also contribute to conjunctival and periorbital oedema. Non‐invasive ventilation may also contribute to ocular surface irritation or injury through air leakage around the mask, even in patients who are not placed in the prone position (Brill 2014). Similar ocular abnormalities may therefore occur in mechanically ventilated patients who have not undergone prone positioning.

Prone positioning may further aggravate this pre‐existing ocular vulnerability through several additional mechanisms. A gravity‐dependent head position can impair venous and lymphatic drainage from the face and orbit, thereby increasing conjunctival and periorbital oedema (Edgcombe et al. 2008; Kwee et al. 2015; Sanghi et al. 2021). Inappropriate head support or direct pressure on the orbit may increase local compression, particularly in the dependent eye. As conjunctival oedema progresses, swollen tissue may protrude through the palpebral fissure and interfere with complete eyelid closure, increasing corneal exposure. Prone positioning should therefore be regarded as a potential additional or aggravating exposure rather than the sole cause of ocular injury in critically ill patients.

Changes in intraocular pressure should also be distinguished from clinical ocular surface complications. Saran et al. (2019) and Deol et al. (2020) reported increases in intraocular pressure during or following prone positioning, particularly with prolonged positioning and in the dependent eye. These findings may reflect impaired venous return, positional hydrostatic effects and local pressure. However, an increase in intraocular pressure does not necessarily indicate that a patient has developed a clinical ocular complication, and the long‐term clinical significance of transient elevations remains uncertain. Future studies should combine serial intraocular pressure measurements with structured ocular examinations and patient‐centred outcomes to clarify whether these physiological changes contribute to clinically meaningful ocular injury.

Among the factors examined in the included studies, lagophthalmos appears to have the clearest clinical relevance to ocular surface injury. Han et al. (2025) identified lagophthalmos, deeper sedation, higher positive end‐expiratory pressure, longer prone‐positioning duration, more frequent prone sessions, and increased ocular secretions as factors associated with exposure keratopathy. Lagophthalmos directly increases corneal exposure and accelerates tear evaporation, whereas sedation is more appropriately interpreted as an indirect contributor through suppression of blinking and impairment of eyelid closure. It should not be regarded as a direct cause of ocular injury in isolation. Clinically, assessment of eyelid closure may therefore provide a more immediate and actionable indicator of ocular risk than the presence of sedation alone.

The reported association between higher positive end‐expiratory pressure and exposure keratopathy should also be interpreted cautiously. Higher PEEP may elevate intrathoracic pressure and reduce venous return from the head and neck, potentially contributing to facial and periorbital congestion (Luecke and Pelosi 2005; Sanghi et al. 2021). When combined with the dependent position of the head, this may worsen chemosis and indirectly impair eyelid closure. However, the current evidence is based on a limited number of studies and does not establish a definitive causal threshold. Because PEEP is determined by respiratory status and cannot be reduced solely to prevent ocular complications, nursing management should focus on identifying patients exposed to higher ventilation pressures and strengthening non‐pharmacological preventive measures, such as avoiding direct orbital compression, maintaining appropriate head alignment, and monitoring facial and ocular oedema.

The duration and frequency of prone positioning may also influence ocular risk (Han et al. 2025). Prolonged sessions increase the duration of dependent venous congestion, ocular exposure and pressure on facial tissues, while repeated sessions may allow cumulative injury to develop before complete recovery has occurred. Nevertheless, the existing studies used different prone‐positioning schedules and assessment time points, and no consistent dose–response threshold has been established. It is therefore premature to recommend a universal ocular assessment interval based solely on the findings of this review. Instead, reassessment should be individualised according to prone‐position duration, eyelid closure, ocular surface appearance, ventilation requirements and the presence of facial oedema.

These findings have several implications for critical care nursing. Ocular assessment should be incorporated into the safety checks performed before, during, and after prone positioning. At minimum, assessment should include eyelid closure, conjunctival oedema or hyperaemia, ocular discharge, corneal clarity, and evidence of direct facial or orbital pressure. Preventive care should aim to maintain eyelid closure, reduce ocular surface exposure, avoid inappropriate pressure, and preserve the effectiveness of protective eye coverings or moisture‐retaining barriers. The choice of eye‐care product should be guided by local protocols, the patient's ocular condition, and the duration of prone positioning rather than by a single universal approach (Demirel et al. 2014; Kocaçal Güler et al. 2011; Koroloff et al. 2004).

Nurses are well placed to identify early changes because they undertake repeated bedside assessments and are directly involved in positioning, eye care and handover. Documentation of eyelid closure, ocular appearance, protective measures and changes observed during prone sessions may improve continuity of care. Escalation for ophthalmological assessment should be considered when routine nursing measures do not resolve eyelid closure problems or when patients develop corneal opacity, persistent epithelial injury, marked chemosis, suspected infection or other signs of potentially vision‐threatening injury. Fluorescein staining may support the early detection of corneal epithelial damage in selected high‐risk patients (Wolffsohn et al. 2017), but its routine use should depend on staff competence, local scope of practice and the availability of ophthalmological support.

The review also highlights important gaps in the evidence. Few studies used standardised baseline ocular examinations, and only a limited number performed multivariable analyses of associated factors. Nursing‐related variables, including eye‐care frequency, type of protective barrier, head position, pressure‐relieving devices and adherence to eye‐care protocols, were inconsistently reported. Future prospective studies should clearly document ocular status before the first prone session, use validated assessment criteria, distinguish newly developed ocular injuries from pre‐existing abnormalities and report outcomes separately for prone and non‐prone patients. Standardised reporting would allow more reliable comparisons and support the development of evidence‐based ocular care protocols for prone‐positioned patients.

### 4.1 Study Limitations

This review has several limitations. First, substantial clinical and methodological heterogeneity across the included studies precluded meta‐analysis. Differences in study populations, prone‐position exposure, ocular outcome definitions, assessment methods and measurement time points also limited direct comparisons between studies. Second, most included studies used observational, non‐comparative or cross‐sectional designs with relatively small samples, which increased the risk of selection bias, information bias and residual confounding. Third, baseline ocular status was not consistently assessed or reported. Consequently, it was not always possible to determine whether the observed ocular findings were newly developed during prone positioning or were already present before study enrolment.

Several studies included mixed intensive care populations and did not provide prone‐position‐specific denominators or outcome estimates. Therefore, the contribution of prone positioning could not always be separated from the underlying effects of critical illness, mechanical ventilation, sedation and other concurrent treatments. In addition, nursing‐related variables, including eyelid‐closure assessment, head position, frequency of eye care, type of protective barrier and adherence to ocular‐care protocols, were incompletely reported, limiting the evaluation of specific preventive interventions.

Finally, only studies published in English were included. This may have introduced language bias and resulted in the omission of relevant evidence published in Chinese or other languages. The findings should therefore be interpreted cautiously.

## 5 Conclusion

Available evidence suggests that critically ill adults exposed to prone positioning may develop ocular abnormalities, particularly chemosis, exposure keratopathy and increased intraocular pressure. However, substantial heterogeneity in study designs, assessment methods and outcome reporting limits direct comparison of the findings. Critical care nurses should incorporate ocular risk assessment into prone‐positioning care, with attention to eyelid closure, ocular surface exposure, facial or conjunctival oedema and direct orbital pressure. Preventive eye care, early identification of abnormalities and timely ophthalmological referral are important for reducing potential ocular injury. Further prospective studies using standardised baseline examinations and prone‐position‐specific outcome reporting are needed.

## Author Contributions

**Zongheng Jiang:** conceptualization, methodology, investigation, data curation, formal analysis, writing – original draft. **Peng He:** writing – review and editing. **Peipei Gu:** methodology, investigation, data curation, formal analysis, writing – review and editing, validation. **Yue Jiang:** writing – review and editing. **Jianming Wang:** writing – review and editing. **Fei Zeng:** conceptualization, methodology, formal analysis, validation, supervision, project administration, writing – review and editing.

## Funding

The authors have nothing to report.

## Disclosure

Protocol Registration: The protocol for this systematic review was registered in PROSPERO (registration number: CRD420251268507). Permissions to Reproduce Material: All figures and tables were prepared by the authors; therefore, permission to reproduce material was not required.

## Ethics Statement

The authors have nothing to report.

## Consent

Informed consent was not required because this review did not collect individual‐level patient data or involve direct contact with human participants.

## Conflicts of Interest

The authors declare no conflicts of interest.

## Supporting information


**Table S1:** JBI critical appraisal of the included cross‐sectional studies.
**Table S2:** Methodological quality assessment of the included cohort and retrospective record‐review studies using the Newcastle–Ottawa Scale.
**Table S3:** Methodological quality assessment of the included non‐comparative study using the MINORS instrument.

## Data Availability

All data analysed in this systematic review were obtained from previously published studies. The data supporting the findings of this review are available within the article and its Supporting Information.
