# Supplementary material for: Ocular Complications During Prone Positioning Ventilation in Critically Ill Adults: A Systematic Review
Source: Nurs Open. 2026 Aug 21;13(8):e70770. doi: 10.1002/nop2.70770 (PMC13494851; doi:10.1002/nop2.70770)
Supplement: Supplementary file 1 [file NOP2-13-e70770-s001.docx]

Supplementary Material

Table S1. JBI critical appraisal of the included cross-sectional studies

| **Study** | **Q1** | **Q2** | **Q3** | **Q4** | **Q5** | **Q6** | **Q7** | **Q8** | **Q9** | **Overall Appraisal** |
| --- | --- | --- | --- | --- | --- | --- | --- | --- | --- | --- |
| Han et al. (2025) | Y | Y | Y | Y | N | Y | Y | Y | Y | High (8/9) |
| Ceylan et al. (2022) | Y | Y | Y | U | N | Y | Y | Y | Y | Moderate (7/9) |
| Ekici Gok et al. (2021) | Y | Y | Y | N | N | Y | U | Y | Y | Moderate (6/9) |

Note: Y, yes; N, no; U, unclear. Q1–Q9 represent the items of the JBI critical appraisal checklist for studies reporting prevalence data. Studies scoring 7–9 were considered high quality, 4–6 moderate quality and 1–3 low quality.

Table S2. Methodological quality assessment of the included cohort and retrospective record-review studies using the Newcastle–Ottawa Scale

| **Study** | **S1** | **S2** | **S3** | **S4** | **C1** | **C2** | **O1** | **O2** | **O3** | **Total Stars** | **Quality Level** |
| --- | --- | --- | --- | --- | --- | --- | --- | --- | --- | --- | --- |
| Saran et al. (2019) | ★ | - | ★ | ★ | - | - | ★ | ★ | ★ | 6/9 | Moderate–high |
| Deol et al. (2020) | - | - | ★ | ★ | - | - | ★ | ★ | ★ | 5/9 | Low–moderate |
| Carvalho et al. (2023) | ★ | - | ★ | ★ | - | - | - | ★ | ★ | 5/9 | Low–moderate |

Note: ★ indicates that the criterion was met; – indicates that the criterion was not met. S, selection; C, comparability; O, outcome. Because the included studies were primarily single-group studies without a non-exposed cohort, several comparability and selection items were not applicable, which limited the maximum achievable score.

Table S3. Methodological quality assessment of the included non-comparative study using the MINORS instrument

| **Study** | **Q1** | **Q2** | **Q3** | **Q4** | **Q5** | **Q6** | **Q7** | **Q8** | **Total Score** | **Quality Level** |
| --- | --- | --- | --- | --- | --- | --- | --- | --- | --- | --- |
| Bajwa et al. (2010) | 2 | 2 | 0 | 2 | 0 | 2 | 2 | 0 | 10/16 | Moderate |

Note: Q1, clearly stated aim; Q2, inclusion of consecutive patients; Q3, prospective data collection; Q4, endpoints appropriate to the study aim; Q5, unbiased assessment of endpoints; Q6, follow-up period appropriate to the study aim; Q7, loss to follow-up below 5%; Q8, prospective sample-size calculation. Each item was scored 0 if not reported, 1 if reported but inadequate and 2 if reported and adequate. The maximum score for a non-comparative study was 16.
